# Inhibitors Targeting CDK9 Show High Efficacy against Osimertinib and AMG510 Resistant Lung Adenocarcinoma Cells

**DOI:** 10.3390/cancers13153906

**Published:** 2021-08-03

**Authors:** Jaya Padmanabhan, Biswarup Saha, Chase Powell, Qianxing Mo, Bradford A. Perez, Srikumar Chellappan

**Affiliations:** 1 Department of Tumor Biology, H. Lee Moffitt Cancer Center and Research Institute, 1209 USF Magnolia Drive, Tampa, FL 33612, USA; Biswarup.Saha@moffitt.org (B.S.); Srikumar.Chellappan@moffitt.org (S.C.); 2 Department of Radiation Oncology, H. Lee Moffitt Cancer Center and Research Institute, 1209 USF Magnolia Drive, Tampa, FL 33612, USA; Chase.Powell@moffitt.org (C.P.); Bradford.Perez@moffitt.org (B.A.P.); 3 Department of Biostatistics and Bioinformatics, H. Lee Moffitt Cancer Center and Research Institute, 1209 USF Magnolia Drive, Tampa, FL 33612, USA; Qianxing.Mo@moffitt.org

**Keywords:** transcriptional CDKs, BRD4, EGFR, K-Ras_G12C_, lung cancer organoids

## Abstract

**Simple Summary:**

Non-small cell lung cancer accounts for 80% of all lung cancer cases. While a subset of non-small cell lung cancer patients respond to immunotherapy, those who are treated with chemotherapy or targeted therapy develop resistance to the drugs. Thus, novel therapeutic strategies are needed to combat this disease. Here we show that inhibitors of the cyclin-dependent kinase 9 are highly effective in preventing the growth of a variety of lung cancer cell lines and lung cancer organoids with high potency. These inhibitors suppressed the expression of several genes like Sox2, Sox9, and Mcl1 that promote tumor growth, facilitating growth arrest. Since inhibitors of cyclin-dependent kinase 9 are undergoing clinical trials for hematological malignancies, our studies suggest that these inhibitors would be attractive candidates to combat non-small cell lung cancer.

**Abstract:**

Non-small cell lung cancer has a 5-year survival rate of less than 12–15%, calling for the development of additional therapeutic strategies to combat this disease. Here we tested the efficacy of inhibiting cyclin-dependent kinase 9 (CDK9) on lung cancer cell lines with K-Ras and EGFR mutations and on lung cancer organoids. Three different CDK9 inhibitors reduced the viability and anchorage-independent growth of lung cancer cell lines at very low nanomolar to micromolar concentrations. CDK9 inhibition suppressed the expression of the anti-apoptotic protein, Mcl1, as well as the embryonic stem cell transcription factors, Sox2 and Sox9, which are pro-tumorigenic. In contrast, treatment with CDK9 inhibitors increased the levels of WT p53 and its downstream target p21 in K-Ras mutant cell lines. Furthermore, the CDK9 inhibitors could markedly reduce the viability of Osimertinib-resistant PC9 and AMG510-resistant H23 and H358 cells with comparable efficacy as the parental cells. CDK9 inhibitors could also significantly reduce the growth and viability of lung cancer organoids with high potency. Taken together, the data presented here strongly suggest that CDK9 inhibitors would be efficacious against K-Ras mutant and EGFR mutant NSCLCs, including those that develop resistance to targeted therapies.

## 1. Introduction

Non-small cell lung cancer (NSCLC) accounts for approximately 85% of all lung cancers and causes the highest number of cancer-related mortalities in the US [1]. NSCLCs are predominantly driven by mutations in the K-Ras and EGFR oncogenes, with mutations in K-Ras more prevalent in smokers [2]. Targeted therapies and immunotherapy using checkpoint inhibitors have considerably improved the overall outcome of lung cancer patients in recent years [3,4,5,6,7,8,9]. At the same time, only a subset of the patients benefit from immunotherapy [10,11,12,13,14,15], while patients treated with targeted therapies, such as EGFR inhibitors, invariably develop resistance to the agents [16,17,18,19]. Therefore, additional strategies need to be developed to effectively treat the patients who do not respond to these therapies and those who develop resistance to the targeted therapeutic agents.

Drug-resistance develops through various mechanisms, including acquisition of secondary mutations (T790M gatekeeper mutation in EGFR inhibitor treated patients), activation of compensatory pathways (amplification of c-Met), induction of stemness-inducing factors (YAP1, Sox2, and Sox9), acquisition of mesenchymal phenotype via EMT, or acquisition of a small-cell lung cancer phenotype [20,21,22,23,24]. Given this background, drugs that can concomitantly target multiple pro-tumorigenic pathways within the tumor niche can be expected to be more effective in preventing tumor growth and overcoming drug resistance. In this regard, we hypothesized that targeting CDK9, the transcription-associated cyclin-dependent kinase, would be highly efficacious in combating NSCLCs harboring different gene mutations.

Oncogene addiction of cancer cells have been studied extensively, which shows the dependence of cancer cells on the activity of the driver mutation for tumor growth [25,26,27]. It has been established that oncogenic mutations invariably result in altered signaling events, which impact the overall transcriptional and metabolic dependencies of cancer cells. Changes in transcriptional activity that promote the proliferation and survival of cancer cells are driven by genetic or epigenetic events, which eventually lead to addiction of the cancer cells to altered or enhanced transcription [28,29]. Efforts to target transcriptional addiction have resulted in the development of agents that affect the activity of specific transcription factors, chromatin remodeling proteins, and components of the general transcriptional machinery [30].

In this context, inhibitors targeting the transcription-associated CDKs (including CDK7, CDK8, CDK9, CDK12, and CDK13) have gained attention as attractive candidates to suppress tumor-specific signaling cascades [31,32,33,34,35]. In addition to their role in regulating transcription, these CDKs play an essential role in the assembly and activity of super-enhancers (SE) and can thus modulate the levels of a wide array of genes/gene products that regulate oncogenesis [36]. CDK9, which phosphorylates the carboxy-terminal domain (CTD) of RNA Pol II, plays an important role in promoting basal and induced gene expression in various cell systems, promoting cell proliferation and survival [37].

CDK9 is a component of the positive transcription elongation factor b (P-TEFb-CDK9/CyclinT1) complex, which phosphorylates the RNA Pol II CTD at Ser-2, allowing productive elongation and generation of long transcripts from which mRNAs are derived [38,39,40]. Though it was thought that CDK9 is mainly involved in transcriptional elongation, lately it has been shown to play a role in RNA Pol II pause–release, elongation, maturation, and termination steps in transcription [37]. Further, CDK9 was found to regulate the expression of c-Myc and Mcl1, which are known to promote cell proliferation and survival, and CDK9 inhibition has been shown to interfere with c-Myc function in K-Ras mutant pancreatic cancers and hematologic cancer cells [41,42,43,44,45,46,47,48,49]. Given this background, we hypothesized that inhibitors of CDK9 would be effective in combating the growth of NSCLC cells. The results presented here show that CDK9 inhibitors suppress the growth of NSCLC cell lines and tumor organoids with high potency, with IC_50_s in the nanomolar range; CDK9 inhibitors were also effective against cell lines that were rendered resistant to the third-generation EGFR inhibitor Osimertinib [50,51,52], as well as the K-Ras_G12C_ inhibitor, AMG510 (Sotorasib) [53,54]. Western blot analysis showed that CDK9 inhibition led to the suppression of Sox2, Sox9, and Mcl1, which are known to contribute to stemness and survival, respectively. Further, CDK9 has been suggested to play a role in maintaining gene silencing at heterochromatin loci, thus suppressing the expression of tumor suppressive genes [55]. Supporting this contention, inhibition of CDK9 enhanced the levels of p53 in NSCLC cell lines that harbor wild type p53. These findings raise the possibility that targeting CDK9 would be a viable and practical strategy to combat NSCLC.

## 2. Materials and Methods

Materials: F12-K and RPMI medium were purchased from Invitrogen/Gibco (Carlsbad, CA, USA); heat inactivated FBS was from VWR Scientific (Atlanta, GA, USA); SNS032, LY2857785, AZD4573, JQ1, Osimertinib, and AMG510 were from Selleck Chemicals (Huston, TX, USA). MTT reagent was from Sigma (St. Louis, MO, USA). Antibodies to RNA Pol II 3E7C7 that detects Ser-2 phosphorylation was from Novus Bio (Littleton, CO, USA), RNA Pol II CTD4H8 that detects Seine 5 phosphorylated Pol II, Mcl1 and p53 antibodies were from Santa Cruz Biotechnology (SCBT, Dallas, TX, USA); antibodies to c-Myc, SOX2, SOX9, and PARP were from Cell Signaling Technologies (Danvers, MA, USA), and GAPDH antibody was from Sigma (St. Louis, MO, USA). Alexa Fluor 488 conjugated secondary antibody, HRP-conjugated secondary antibodies, and the majority of the western blotting reagents and ECL solution were purchased from Thermo Fisher (Waltham, MA, USA). Mounting medium with DAPI was from Vector Laboratories (Burlington, CA, USA). FITC Annexin V apoptosis detection kit with PI was from BioLegend (San Diego, CA, USA). Materials used for culturing the lung tumor organoids (R-spondin 1, FGF7, FGF10, Noggin, A83-01, Y-27632, SB202190, and Nicotinamide) were mainly purchased from PeproTech (Cranbury, NJ, USA), B27 supplement, GlutaMAX, advanced DMEM/F12, and Penicillin/Streptomycin were from Invitrogen (Carlsbad, CA, USA), primocin was from InvivoGEN (San Diego, CA, USA), basement membrane matrix (BME type II) was from Trevigen (Gaithersburg, MD, USA), and N-acetylcysteine was from Sigma (St. Louis, MO, USA).

*NSCLC cell lines*: K-Ras mutant A549 (ATCC CCL-185), H460 (HTB-177) and H23 (CRL-5800) and EGFR mutant H1650 (CRL-5883) and H1975 (CRL-5908) cells were purchased from ATCC (Gaithersburg, MD, USA). PC9 cells were purchased from Sigma (90071810, formerly known as PC14). A549 cells were grown in F12-K medium supplemented with 10% FBS and 1% antibiotic-antimycotic, and the rest of the cells were cultured in RPMI supplemented with 10% FBS and 1% antibiotic-antimycotic. Resistant cells used in this study were generated by dose escalation method with Osimertinib (20 nM to 4 µM in the case of PC9 cells) or AMG-510 (0.5 µM to 15 µM in the case of H23 and 0.1 μM to 5 μM in the case of H358 cells) for 4 months, after which the cells were maintained in 4 µM Osimertinib or 15 µM AMG510, respectively.

*Lung Tumor Organoids*: organoids were generated from patient-derived tumor tissue samples following the protocol from Hans Clever’s group [56]. Briefly, solid lung tissues were minced and washed with advanced DMEM/F12 containing 1× GlutaMAX, 10 mM HEPES, and antibiotics prior to digestion with collagenase on an orbital shaker at 37 °C for 1–2 h. Digested tissue was sheared with fire-polished Pasteur pipettes and strained through a 100 µM filter. The strained suspension was mixed with 2% FCS and centrifuged to collect the pellet. Any red blood cells were removed by treating with the red blood lysis buffer from Roche for 5 min. The pellets were resuspended in the advanced DMEM/F12 medium containing GlutaMAX and HEPES and spun to collect the cells. The cell pellet was resuspended in Cultrex growth factor reduced BME type 2 Matrigel, 40 µL of the suspension was allowed to solidify in pre-warmed 24 well low attachment suspension culture plates at 37 °C for 15 min. After the gelation, 400 µL medium was added and the plates transferred to humidified CO_2_ incubators. The organoid medium contained agents that activate or inhibit various signaling pathways, such as R-Spondin, FGF7, FGF10, B27 supplement, and Noggin (activating agents), and A83-01, Y27632, SB202190, (signaling inhibitors), along with *N*-acetylcysteine (antioxidant), nicotinamide, and antibiotic/antimycotic in advanced DMEM/F12. Medium was changed every 4th day and organoids were passaged every 2 weeks. The lung cancer tumor organoid model (154838) utilized for these experiments is derived from a patient with poorly differentiated lung carcinoma. Tumor and organoid sequencing with QIAseq Targeted Cancer Gene Panel confirmed mutations in multiple commonly mutated oncogenes and tumor suppressors including *TP53* and *APC*, which were found to have nonsense mutations leading to silenced gene expression. Additionally, a urothelial carcinoma metastatic to lung (135123) was collected and tumor organoids were derived. This tumor model was confirmed to have activating *HRAS* Q61R mutation by WES sequencing present, in both the original tumor and the tumor organoid.

*Cell Viability and IC_50_ determination:* viability of the various cell lines was measured using MTT (3-(4, 5-dimethylthiazol-2yl)-2,5-diphenyl tetrazolium bromide) assays [57,58]. A total of 2500 cells were cultured per well in 96-well plates in 100 µL medium and treated with at least 10 different concentrations, ranging from 10 nM to 40 µM of SNS032 and LY2857785, or 3 nM to 2 µM of AZD4573, alone or in combination with 0.5 µM JQ1, for 72 or 96 h. Moreover, 10 µL of MTT solution (5 mg/mL) was added to each well and plates were incubated for 2–4 h, the formazan crystals were solubilized in 100 µL DMSO prior to measurement of absorbance at 570 nm and IC_50_ analysis was performed using GraphPad Prism software for graphing and statistics. Viability of the Organoids was conducted using the CellTiter-Glo luminescent cell viability assay kit from Promega (Madison, WI, USA). Organoids were dissociated (1000 cells/well in suspension) and were cultured for 48 h in ultralow attachment 96 well plates prior to treatment with the CDK9 inhibitors for 96 h. At the end of treatment, CellTiter-Glo reagent was added to the wells and viability was determined by measuring the luminescence on a 96-well formatted luminometer.

*Apoptosis detection by Annexin V Staining:* for detection of apoptosis in cells treated with CDK9 inhibitors we used the FITC Annexin V apoptosis detection kit with PI from BioLegend, following the manufacturer’s instructions. Briefly, A549, H1650, PC9 parent and osimertinib-resistant, and H23 parent and AMG510-resistant cells were plated into 8 chamber slides at a density of 15,000–20,000 cells/well. After 24 h cells were treated with 20 nM AZD4573, 0.5 µM JQ1 or a combination of AZD4573 and JQ1 for 24 h. Cells treated with DMSO served as control. At the end of the treatment cells were washed twice with cell staining buffer and incubated with 100 µL Annexin V/PI diluted in binding buffer for 15–30 min at room temperature, protected from light. Subsequently, 250 µL of binding buffer was added to the wells and the cells were imaged immediately using an inverted EVOS fluorescent microscope at 20× magnification.

*Western Blot Analysis:* cells cultured on 60-mm tissue culture dishes (2.5 × 10^5^ cells/dish) were treated with 20 nM AZD4573 for the indicated times. At the end of the treatment, cells were washed with cold PBS and lysates were prepared in M2 lysis buffer (20 mM Tris (pH 7.6), 0.5% NP40, 250 μM NaCl, 3 μM EGTA, 3 μM EDTA, 5 μM leupeptin, 1 μM DTT, 100 μM PMSF, 500 μM NaF, 5 μM aprotinin, 5 μM pepstatin, 5 μM trypsin/chymotrypsin, and 500 μM Na_2_VO_3_). The lysates were centrifuged at 14,000 rpm for 15 min at 4 °C and supernatants were collected. 30–40 μg of protein was resolved by PAGE and transferred to 0.2 μm nitrocellulose membrane. Membranes were incubated with 5% nonfat dry milk in TBS for one hour to inhibit non-specific binding and incubated with primary antibody overnight at 4 °C. Membranes were washed thoroughly with PBS containing 0.05% Tween 20 (PBST) and incubated with 1:5000 dilution of goat anti-rabbit or goat anti-mouse HRP-conjugated secondary antibodies. After additional washes in PBST, the blots were incubated in ECL reagent and exposed to X-ray film.

*Immunostaining Analysis*: A549 or H460 cells (10,000/well) were cultured on poly D-lysine coated 8-chamber slides for 24 h, and treated with 1 µM SNS032 for 24 h. Cells were fixed with 4% paraformaldehyde and immunostaining analysis for p53 was conducted using the established protocols [59,60]. Alexa Fluor 488 was used for detection of the signals. The slides were mounted using Vectashield anti-fade mounting medium containing DAPI and analyzed by confocal microscopy.

*Colony Formation Assay*: to analyze the effect of the CDK9 inhibitors on anchorage independent growth of lung cancer cells, soft agar colony formation assay was performed, as per our published protocols [58]. Briefly, a base layer was generated by placing 2 mL of medium containing 0.6% agar in 12 well plates. Once the base layer is solidified, 1 mL of medium containing 0.3% agar, 5000 cells (A549 or H460), and the appropriate inhibitor concentrations was layered on top of the base layer. After the top agar was solidified, the plates were moved to the CO_2_ incubator. The wells were re-fed with 150 μL medium containing drugs once a week to prevent drying. Each treatment was conducted in triplicates and repeated twice. At the end of two to three weeks, 1 mg/mL MTT solution was added to the wells and incubated for 4 h to overnight for staining and visualization of colonies. The plates were scanned using an Epson scanner and quantified using Image J image analysis program.

*Live/Dead assay using 3,3′-dioctadecyloxacarbocyanine (DiOC_18_) and propidium iodide (PI):* to determine the viability of cells in lung tumor organoids treated with the CDK9 inhibitors we used the live/dead cell-mediated cytotoxicity kit from Thermo Fisher Scientific/Molecular Probes [61,62]. The organoid cultures were treated for 4 days with the indicated CDK9 inhibitors and labelled overnight with DiOC_18_, a membrane stain, as per the protocol provided by the manufacturer. After 24 h, the membrane impermeable propidium iodide, provided in the kit, was added to the culture medium and incubated for 5 min at room temperature. The organoids were observed under an inverted EVOS fluorescent microscope and images were taken at 10× magnification. The green represents live cells with intact membrane and red represents cells with compromised membrane, which allowed uptake of PI, suggesting cell death upon treatment with the CDK9 inhibitors. The experiment was conducted in duplicate for two times.

*TCGA data analysis*: the TCGA RNA-seq gene expression data (version 2016_01_28) were obtained from the FireBrowse portal (http://firebrowse.org/ (accessed on 3 August 2021)). The gene expression was quantified by normalized read counts for mRNA. Two sample *t*-test was used to compare the expression of CDK9, SOX2, and SOX9 between the tumor tissues (n = 515) and normal tissues (n = 59). R (v 4.0.3) was used for statistical analysis and making graphs.

*Statistical analysis*: all experiments were conducted at least three independent times unless otherwise mentioned, viability assay and soft agar assay were conducted in triplicates each time and statistical analysis was performed using *Student’s t test*. GraphPad Prism software for graphing and Statistics was used for IC_50_ analysis.

## 3. Results

### 3.1. K-Ras and EGFR-Mutant Lung Cancer Cells Are Sensitive to CDK9 Inhibitors

**Figure 1:**
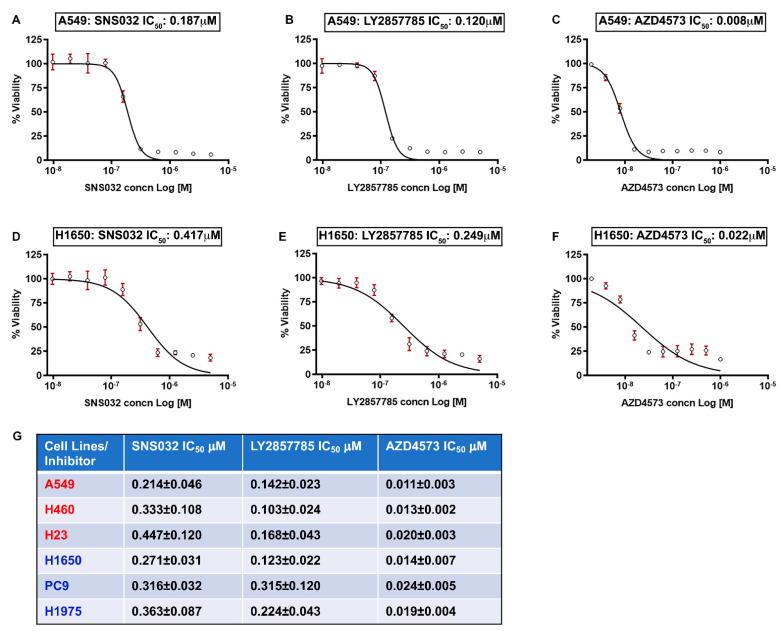
(**A**–**G**) CDK9 inhibitors reduce viability of NSCLC cell lines at very low concentrations: K-Ras mutant A549, H460, or H23 and EGFR mutant H1650, PC9, and H1975 lung cancer cells were treated with 10 different concentrations of the CDK9 inhibitors SNS032, LY2857785, or AZD4573 ranging from (3 nM to 40 µM) for 96 h and an IC_50_ analysis was conducted using the GraphPad Prism software. Representative IC_50_ curves for A549 (**A**–**C**) and H1650 (**D**–**F**) are shown. (**G**) Chart shows the IC_50_ values (mean ± SD) for the CDK9 inhibitors in A549, H460, H23, H1650, PC9, and H1975 cell lines from 3 independent experiments, conducted in duplicates.

Experiments were designed to assess the efficacy of CDK9 inhibitors in reducing the viability of multiple human NSCLC cell lines. The first set of experiments was conducted on three K-Ras mutant (A549, H460, and H23) and three EGFR mutant (H1650, PC9 and H1975) lung adenocarcinoma cell lines. The IC_50_ for growth suppression was determined using 10 different concentrations of inhibitors, ranging from 10 nM to 40 µM. Cells were treated with three different CDK9 inhibitors for 96 h and viability was measured by MTT assay. It was found that all three CDK9 inhibitors tested, namely SNS032, LY2857785, and AZD4573, could markedly reduce the viability of K-Ras mutant A549, H460, and H23 and EGFR mutant H1650, PC9, and H1975 cells with high potency. AZD4573 showed the most efficacy, with IC_50_ values ranging from 8 to 27 nM; LY2857785 and SNS032 showed IC_50_s below 0.5 µM in these cell lines. Representative IC_50_ curves from one of the K-Ras mutant and one of the EGFR mutant cell lines is shown in Figure 1 (A–C: A549 and D–F: H1650; and G: Chart—showing the IC_50_ values for the three different inhibitors in the six cell lines mentioned here). These experiments suggest that CDK9 inhibition is highly effective in reducing the viability of NSCLC cells.

### 3.2. CDK9 Inhibition Suppresses Sox2, Sox9, and Mcl1 and Elevates p53 Protein Expression

**Figure 2:**
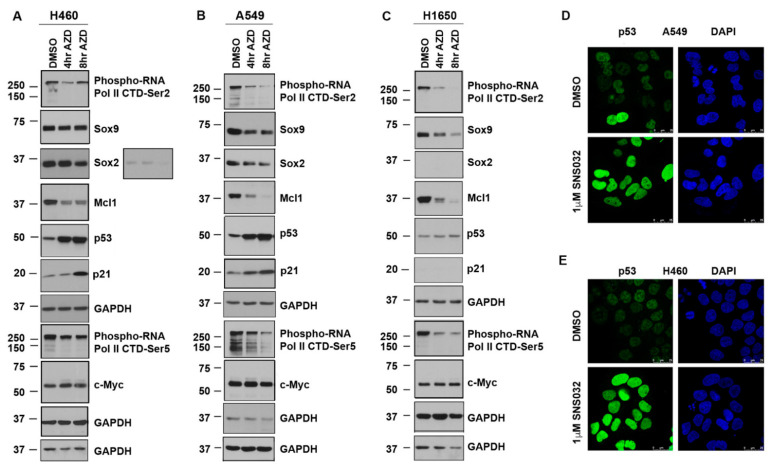
CDK9 inhibitors suppress Mcl1, Sox2, and Sox9 and enhance p53 expression in lung cancer cells: (**A**–**C**) H460, A549, and H1650 cells were treated with AZD4573 at 20 nM for 4 and 8 h and samples were analyzed by western blotting. Blots probed with the Phospho-Ser2-RNA Pol II CTD antibody show a decrease in the phosphorylation levels in A549 and H1650 cells, and to a lesser extent in H460 cells, indicative of CDK9 inhibition. Ser5-specific phosphorylation also showed a decrease in H460, A549, and H1650 cells, with H460 showing lesser effect. AZD4573 treatment led to a reduction in the levels of Mcl1 in H460, A549, and H1650 cells. Similarly, AZD4573 treatment showed a better reduction in the expression of Sox9 in A549 and H1650 cells compared to H460 cells. Sox2 reduction was more visible in A549 than H460 (both dark and light exposure for Sox2 in H460 is shown); H1650 did not show any detectable levels of Sox2. Analysis of the blots using a p53 antibody showed marked increase in its levels in H460 and A549 cells; H1650 did not show any visible changes. Both H460 and A549 cells also showed an increase in p21 levels. AZD4573 did not affect the levels of c-Myc in the cells. Blots were re-probed with GAPDH antibody for normalization. (**D**) A549 and (**E**) H460 cells were treated with SNS032 at 1 µM for 24 h and immunofluorescence analysis was conducted using a p53 antibody. Results show a drastic increase in p53 levels upon treatment with CDK9 inhibitor compared to the DMSO vehicle treated cells.

Western blotting was conducted to assess the molecular changes brought about by the inhibitors in the NSCLC cell lines. A549, H460, and H1650 cells were treated with 20 nM AZD4573 for 4 and 8 h. Lysates were prepared and western blotting was performed as per our published protocols [24] (Figure 2A). As expected, there was a marked reduction in the levels of RNA Pol II CTD phosphorylation at Ser2, which persisted in A549 and H1650 cells; the levels appeared to recover at later time points in H460 cells (Figure 2A–C, top panel). Probing the blots with a Ser5-specific Phospho-RNA Pol II antibody showed that phosphorylation at this site is also reduced (Figure 2A–C, panel 8). These changes in CTD phosphorylation indicate a reduction in Pol II-mediated transcription. Since Mcl1 expression is known to be regulated by CDK9 [41], we analyzed the levels of this protein in cells treated with AZD4573; we observed a drastic reduction in Mcl1 levels in all three cell lines, with H460 showing less pronounced effect compared to A549 and H1650 cells (Figure 2A–C, panel 4). Interestingly, treatment with AZD4573 increased the levels of p53 in H460 and A549 cells, compared to control DMSO-treated cells (Figure 2A,B, panel 5); H1650 cells did not show any noticeable change (Figure 2C, panel 5). Levels of the CDK inhibitor, p21, which is an established p53 target, were also increased in AZD-treated H460 and A549 cells (Figure 2A,B—Panel 6). Levels of the embryonic stem-cell transcription factors, Sox2 and Sox9, were reduced in both H460 and A549 cells whereas H1650 cells showed a marked reduction in Sox9; Sox2 was below detection level (Figure 2A–C, Panels 2 and 3). Additionally, we examined the levels of c-Myc protein, since P-TEFb complex is recruited to active promoters by the BET bromodomain protein BRD4, which is known to regulate expression of c-Myc [63,64]. There was no notable change in the levels of c-Myc, although its levels persisted in the cells treated with AZD4573 Figure 2A–C, Panel 9). These results indicate that inhibition of CDK9 might be reducing cell viability by elevating p53 levels while simultaneously suppressing the levels of Mcl1 and the embryonic stem-cell transcription factors Sox2 and Sox9, which are known to promote cancer cell survival. GAPDH antibody was used for normalization of the blots. Immunofluorescence analysis conducted on A549 and H460 cells, treated for 24 h with 1 µM SNS032, showed a notable increase in the nuclear p53 levels, confirming that CDK9 inhibition enhances the expression of p53 tumor suppressor in K-Ras mutant NSCLC cells (Figure 2D,E).

### 3.3. Combined Inhibition of CDK9 and BRD4 Markedly Reduces Cell Viability

**Figure 3:**
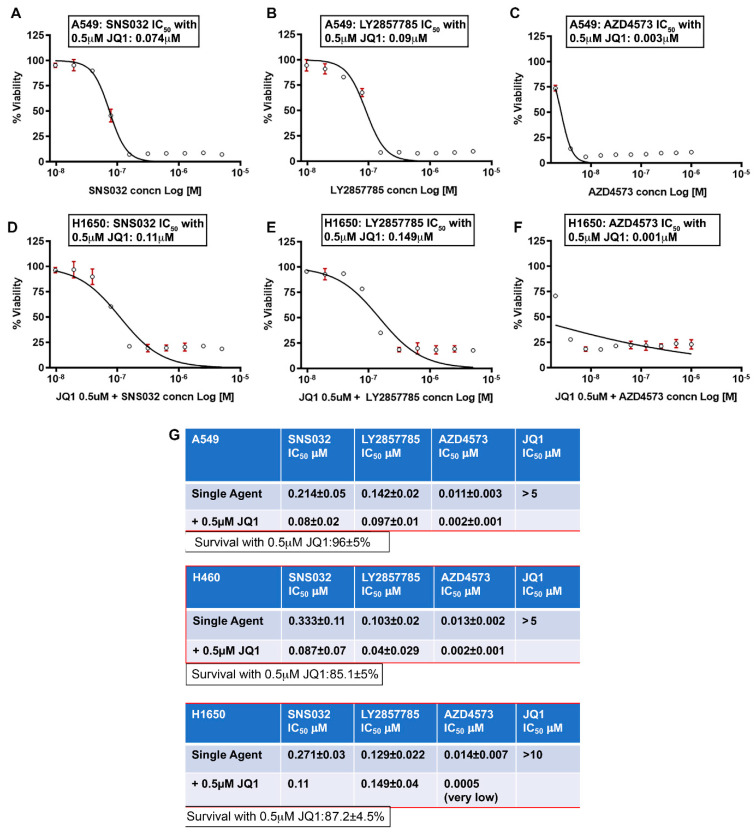
BRD4 inhibitor JQ1 enhances the sensitivity of CDK9 inhibitors on lung cancer cells: (**A**–**F**) representative IC_50_ curves for CDK9 inhibitors with JQ1: A549 (**A**–**C**) and H1650 (**D**–**F**) cells were treated with 10 different concentrations of SNS032, LY2857785, or AZD4573, in the presence of 0.5 µM JQ1, for 96 h, and IC50 analysis was conducted using GraphPad Prism. (**G**) Chart shows the IC_50_ values (mean ± SD) for SNS032, LY2857785, or AZD4573 alone or in combination with 0.5 µM JQ1 and for JQ1 alone, from three independent experiments conducted in duplicate. JQ1 by itself at 0.5 µM showed very little effect on the cancer cells; more than 85% of the cells were alive.

The above results showed that Sox2 and Sox9, whose expression is regulated by super-enhancers, is suppressed in NSCLC cells upon CDK9 inhibitor treatment. The chromatin remodeling protein BRD4 is an established bromodomain protein that plays a crucial role in regulation of c-Myc. Since BRD4 plays an important role in recruiting the P-TEFb (CDK9/Cyclin T) complex to super enhancers thereby promoting active gene transcription [65], and since BRD4 plays a role in the development of lung cancers [66], we examined if drugs targeting this bromodomain protein improve the efficacy of CDK9 inhibitors. This contention was further supported by the observation that c-Myc levels appeared to persist upon CDK9 inhibitor treatment. We tested if inhibiting BRD4 using JQ1 enhances the efficacy of CDK9 inhibitors. IC_50_ analysis for JQ1 showed that 50% reduction in viability is achieved at concentrations ranging from 5 µM to greater than 10 µM in A549, H460, and H1650 cells. A combination treatment experiment showed that combining a sub-lethal dose of JQ1 (0.5 µM) with SNS032, LY2857785, or AZD4573 significantly reduced the IC_50_ of these CDK9 inhibitors in A549 (Figure 3A–C), H460 (IC_50_ curves are not shown), and H1650 cells (Figure 3D–F). The IC_50_ values showing the comparison between CDK9 inhibitor alone or in combination with 0.5 µM JQ1 for A549, H460, and H1650 cells are shown in the chart (Figure 3G). JQ1 at 0.5 µM showed only a marginal reduction in viability in A549 (96 ± 5% viable), H460 (85 ± 5% viable), and H1650 (87 ± 4.5% viable) cells. This experiment strongly suggests that combined inhibition of CDK9 and BRD4 would be a useful strategy to combat cancers, which show high levels of MYC or other genes, whose expression is regulated by super-enhancers.

### 3.4. Adherence-Independent Growth of Lung Cancer Cells Is Abrogated upon CDK9 Inhibition

**Figure 4:**
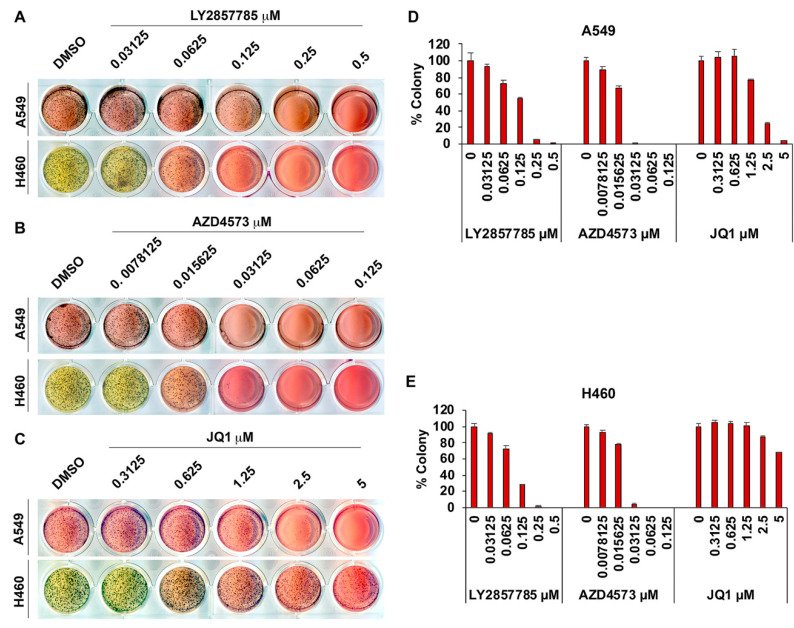
Anchorage-independent growth of lung cancer cells is suppressed by CDK9 inhibitors: (**A**–**C**): soft agar colony formation assay conducted using A549 and H460 cells in the presence of varying concentrations of the CDK9 inhibitors LY2857785 (~31 to 500 nM) and AZD4573 (~8 to 125 nM) or the BRD4 inhibitor JQ1 (~0.3 to 5 µM) show that CDK9 inhibitors effectively inhibit the anchorage-independent growth at low nanomolar concentrations. JQ1 also reduced colony formation at 2.5 µM in A549 cells but not in H460 cells. (**D**,**E**) Bar graphs show quantification of the colonies from A549 (**D**) and H460 (**E**) cells treated with the inhibitors.

The ability to grow in an adherence-independent manner is a notable feature of cancer cells and agents that can prevent adherence-independent growth can be expected to have anti-cancer effects in vivo. Since CDK9 inhibitors could markedly reduce the viability of lung cancer cell lines cultured on tissue culture plates, experiments were conducted to assess if the inhibitors were effective in preventing adherence-independent growth as well. Colony formation in soft agar was used to measure adherence-independent growth of A549 and H460 cells (Figure 4A–E). Treatment with JQ1 could significantly reduce colony formation of A549 cells at 2.5 µM, while H460 cells showed a significant reduction only at concentrations above 5 µM (Figure 4C–E). In striking contrast, the CDK9 inhibitors, LY2857785 and AZD4573, suppressed adherence-independent growth of both the cell lines with notably high potency (Figure 4A,B and the bar graphs in Figure 4D,E). LY2857785 almost completely inhibited colony formation of A549 and H460 cells at 0.25 µM concentration. AZD4573 was even more potent; it completely abrogated adherence independent growth of both A549 and H460 cells at ~31 nM concentration. This suggests that CDK9 inhibition might be a viable and effective strategy to combat NSCLC.

### 3.5. CDK9 Inhibitors Can Eliminate Drug Resistant NSCLC Cells

**Figure 5:**
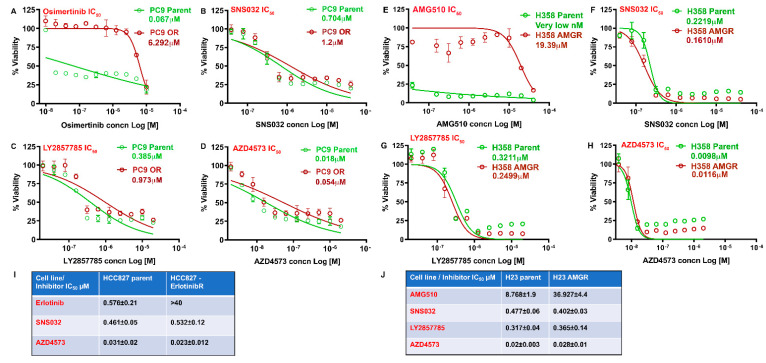
CDK9 inhibitors are effective against Osimertinib-resistant and AMG510-insensitive lung cancer cells: IC_50_ analysis was conducted, on Osimertinib-sensitive and-resistant PC9 cells with varying concentrations of Osimertinib (**A**), or the CDK9 inhibitors SNS032 (**B**), LY2857785 (**C**), or AZD4573 (**D**), using the GraphPad Prism software. (**E**–**H**) IC_50_ curve for AMG510 and CDK9 inhibitors on AMG-sensitive and -insensitive H358 cells treated with ~ AMG510 (**E**), SNS032 (**F**), LY2857785 (**G**), or AZD4573 (**H**). (**I**) Chart shows the IC_50_ values (mean ± SD) for SNS032 and AZD4573 for HCC827 parent and HCC827 erlotinib resistant cells. (**J**) Chart shows the IC50 values (mean ± SD) for SNS032, LY2857785 and AZD4573 on AMG-sensitive and -insensitive H23 cells from three independent experiments conducted in duplicates.

Lung cancer patients treated with EGFR inhibitors inevitably develop resistance, leading to tumor recurrence and mortality. Development of resistance against targeted therapy is common, resulting in the appearance of more aggressive tumors. Hence, we examined if CDK9 inhibitors can eliminate lung adenocarcinoma cells that are resistant to the third-generation EGFR inhibitor, Osimertinib, or the recently developed K-Ras G12C inhibitor, AMG510 (Sotorasib). Towards this purpose, we generated Osimertinib-resistant PC9 cells which harbors an EGFR mutation and AMG510-resistant H23 and H358 cells, which harbors the K-Ras G12C mutation, by dose escalation method. While Osimertinib eliminated the parental PC9 cells with an IC_50_ of ~20 to 70 nM after 72 h of treatment, the resistant cells showed an IC_50_ of greater than 5 µM (more than 70-fold difference) (Figure 5A). All three CDK9 inhibitors, SNS032, LY2857785, and AZD4573, could inhibit the viability of both parent and resistant cells effectively, with the resistant cells showing slightly higher IC_50_ than parent cells (Figure 5B–D). Additionally we examined the effectiveness of the inhibitors on HCC827 EGFR mutant cells that are responsive or resistant to erlotinib. Similar to the results from PC9 cells, both resistant and responsive HCC827 cells were sensitive to the treatment with SNS032 and AZD4573 at very similar concentrations (Figure 5I). In a similar vein, H358 and H23 cells, which were rendered resistant to AMG510, were tested for the efficacy of CDK9 inhibitors. H358 parent cells were very sensitive to the AMG510 treatment and showed an IC_50_ of less than 40 nM. H358 AMG510-resistant cells showed an IC_50_ > 19 µM. IC50 analysis for SNS032, LY2857785, and AZD4573 showed almost identical response on both parental and AMG510-resistant H358 cells (Figure 5E–H). In the case of H23 cells, AMG510 could eliminate the parental cells with an IC_50_ of ~8–10 µM and the resistant cells at ~32–40 µM, a four-fold higher concentration compared to parent cells (Figure 5J, chart). Here also, we found that treatment with SNS032, LY2857785, and AZD4573 could eliminate both the parental and AMG510 resistant H23 cells with comparable IC_50_ values. These experiments suggest that CDK9 inhibitors might be effective in patients who have developed resistance to EGFR and K-Ras-targeted therapies.

### 3.6. Drug-Sensitive and Resistant Lung Cancer Cells Treated with AZD4573 and JQ1 Show Increased Apoptosis and Reduced Mcl1 Expression

**Figure 6:**
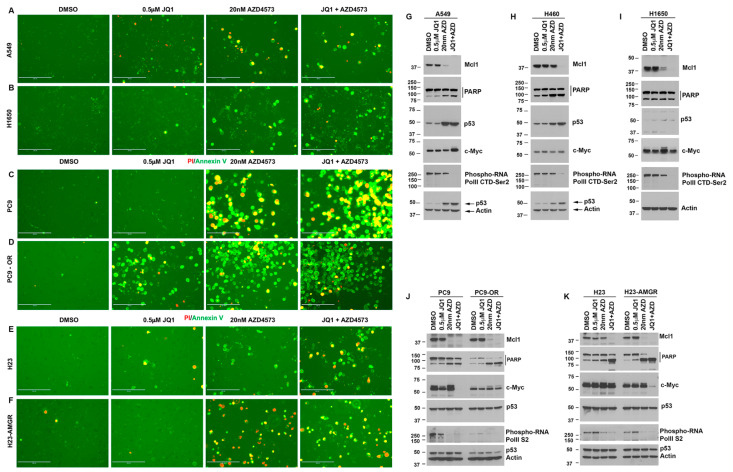
Lung cancer cells treated with AZD4573 and JQ1 show enhanced apoptosis and inhibition of Mcl1: (**A**–**F**) A549 (**A**), H1650 (**B**), PC9 (**C**), PC9-OR (**D**), H23 (**E**), and H23-AMGR (**F**) cells were treated with JQ1, AZD4573, or a combination of both, and stained using Annexin V (green) and PI (red) to determine apoptosis induction. (**G**–**I**) Western blot analysis on A549 (**G**), H460 (**H**), or H1650 (**I**) cells treated with AZD4573, or JQ1 alone, or in combination were analyzed for alterations in Mcl1, PARP, p53, c-Myc or RNA Pol II CTD phosphorylation. Actin was used to re-probe the blots for protein normalization. (**J**,**K**) PC9 cells, sensitive or resistant to osimertinib, and H23 cells sensitive or resistant to AMG510, were treated with JQ1, AZD4573, or their combinations and analyzed with same antibodies as in the case of (**G**–**I**). Results show complete inhibition of Mcl1 and increased PARP cleavage in response to co-treatment with AZD4573 and JQ1.

Since the addition of sublethal concentrations of the BRD4 inhibitor enhanced the efficacy of CDK9 inhibitors on lung cancer cells, we next examined if the combination treatment showed any additive effect on the apoptotic pathway. A549, H1650, PC9 parent or osimertinib resistant, and H23 parent or AMG510 resistant cells were treated with 0.5 µm JQ1, 20 nM AZD4573, or a combination of both, and stained with the apoptosis staining kit containing FITC Annexin V and PI. Results showed that Annexin V positivity was increased in cells treated with AZD4573 alone or in combination with JQ1 (Figure 6A–F). JQ1 by itself showed very little effect at the concentration tested, except in the case of PC9-OR (osimertinib resistant cells), which showed an increase in Annexin V positivity. Both PC9 and H23 cells showed more Annexin V positivity compared to A549 and H1650.

Next, we examined for changes in the anti-apoptotic Mcl1 protein and induction of PARP cleavage in these lung cancer cells. Cells were treated with 0.5 µM JQ1, 20 nM AZD4573, or a combination of both for 24 h; western blotting using Mcl1 and PARP antibodies showed that Mcl1 was completely inhibited by co-treatment in A549, H460 and H1650 cells (Figure 6G–I, top panel). A549 and H460 cells showed an increase in PARP cleavage with AZD4573, and JQ1 addition did not show any drastic increase. H1650 cells on the other hand showed very little change in PARP cleavage with AZD4573 alone or in combination with JQ1 (Figure 6G–I, second panel). Treatment with AZD4573 by itself showed an increase in the levels of p53 in A549 and H460 cells that express WT p53, but not in H1650, which expresses a mutant p53 (Figure 6G–I, Panel 3). Re-probe of the blots with a c-Myc antibody did not show much change in the levels with JQ1 treatment. AZD4573 appeared to show a slight increase in the levels of Myc in H1650 cells and co-treatment reverted this to the basal levels. RNA polymerase II CTD phosphorylation was markedly suppressed in the cells co-treated with AZD4573 and JQ1.

Additionally, we examined if the treatment bring about similar response in the PC9 and H23, parent and drug-resistant cells. As in the case of A549, H460, and H1650, we treated the cells with 0.5 µM JQ1, 20 nM AZD4573, or a combination of both and examined for changes in the proteins mentioned above. Here also there was a complete inhibition of Mcl1 in both parent and resistant cells co-treated with the inhibitors (Figure 6J,K, top panel). Furthermore, we observed a noticeable increase in PARP cleavage in the cells co-treated with the inhibitors (Figure 6J,K, second panel). This agrees with the results from the Annexin V analysis, where we observed a drastic increase in positive cells compared to similarly treated A549 and H1650. C-Myc was suppressed in PC9 parent and H23 AMGR cells co-treated with JQ1 and AZD4573. The levels of p53 were not affected by the treatment, which was expected as these cells express mutant p53. RNA Pol II CTD phosphorylation was also suppressed by the co-treatment on both parent and resistant cells. All together, these results show that CDK9 inhibition can significantly inhibit the growth of lung cancer cells, irrespective of their mutational status, and would help in overcoming the drug-resistance associated with the established therapies.

### 3.7. CDK9 Inhibitors Reduce the Survival of Lung Cancer Organoids

**Figure 7:**
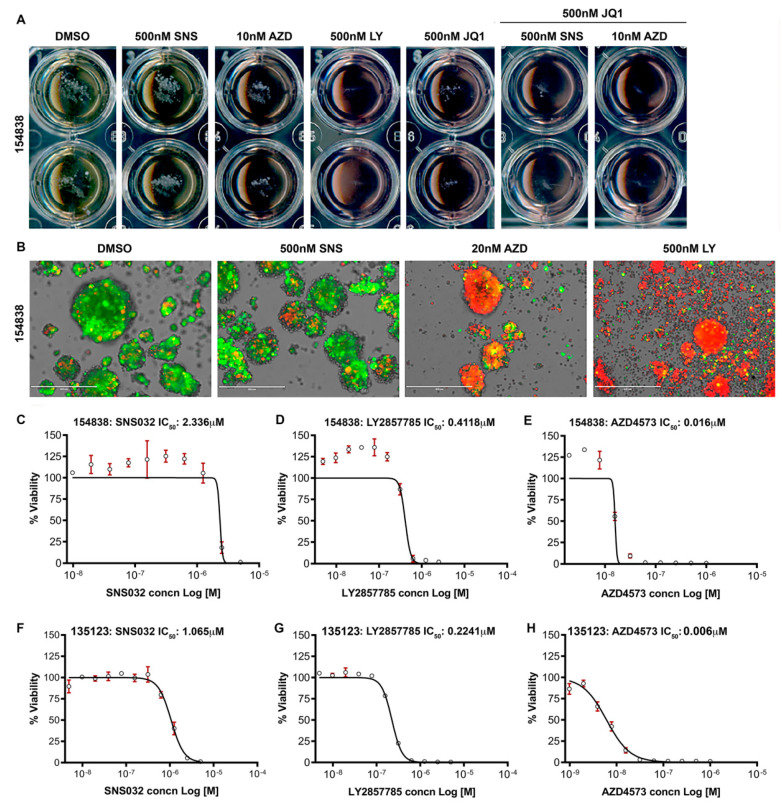
Lung tumor organoids treated with CDK9 inhibitors show drastic reduction in growth and induction of cell death: (**A**) lung tumor organoids (#154838) were treated with 500 nM SNS032 (SNS), 10 nM AZD4573 (AZD), 500 nM LY2857785 (LY), 500 nM JQ1, or JQ1 with SNS032 or AZD4573 for 8 days, and images were generated by scanning the organoid containing plates on a flat-bed Epson scanner. (**B**) Lung tumor organoids (#154838) were stained using the DiOC_18_ green fluorescent probe and propidium iodide (PI) to determine the survival status of cells within the remaining organoid spheres after the CDK9 inhibitor treatment. Green fluorescence is indicative of live cells and PI positivity (red) is indicative of compromised cell membrane and cell death. (**C**–**H**) IC_50_ analysis was conducted on two different lung tumor organoids, #154838 (**C**–**E**) and #135123 (**F**–**H**), with 10 different concentrations of SNS032, LY2857785, or AZD4573 for 96 h, using the GraphPad Prism software.

Patient-derived organoids are notably useful in assessing the anti-cancer effects of drugs, since they recapitulate many of the properties of the parental tumors from which they are derived [67]. Two different lung cancer organoids (#154838 and #135123), established under an IRB-approved protocol, were cultured in specific medium supplemented with the appropriate factors, as described in the Materials and Methods. In the first set of experiments, #154838 organoids were treated for 8 days with SNS032 or LY2857785 at 500 nM or AZD4573 at 10 nM concentrations. Brightfield microscopy showed that treatment with 500 nM LY2857785 completely eliminated the growth of organoids (Figure 7A). SNS032 showed very minimal effect and AZD4573 showed more potency at the doses tested. Treatment of the organoids with 500 nM of JQ1 showed a noticeable suppressive effect; combining 500 nM JQ1 with 500 nM SNS032 or 10 nM AZD4573 completely prevented the growth of the organoids (Figure 7A). Since some of the wells showed residual organoid spheres, we conducted a Live/Dead assay using DiOC18 and propidium iodide, as described in the materials and methods. At the end of the incubation, the organoids were analyzed under an inverted EVOS fluorescence microscope for viability; green fluorescence represents live cells and red represents PI-stained nuclei, indicative of compromised cell membrane and/or cell death. Our results show that treatment with the CDK9 inhibitors induced cell death in the lung cancer organoids (Figure 7B). Next, we conducted a dose response experiment on two different lung tumor organoids, #154838 and #135123 with SNS032, LY2857785, and AZD4573. Results showed that SNS032 suppressed the growth of #154838 and #135123 tumor organoids with an IC_50_ of 2.336 µM and 1.065 µM, respectively, while LY2857785 suppressed the growth with an IC_50_ of 0.412 µM and 0.224 µM, respectively (Figure 7C–E: #154838 and Figure 7F–H: #135123). AZD4573 was very potent in inhibiting the growth of both organoids and showed an IC_50_ of 6nM and 15nM, respectively, in #154838 and #135123. The results from the tumor organoids imply that the CDK9 inhibitors would be effective in eliminating the heterogeneous tumors in vivo.

### 3.8. TCGA Dataset Shows Alterations in CDK9 and Its Targets

**Figure 8:**
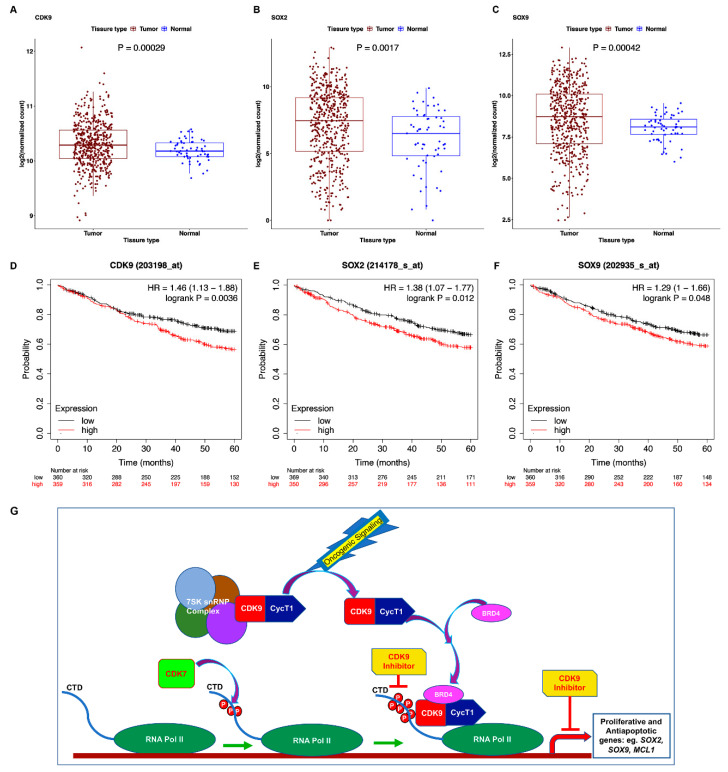
Lung adenocarcinomas show increased expression of CDK9, SOX2, and SOX9: (**A**–**C**) RNAseq dataset from TCGA FireBrowse portal show significantly high expression of CDK9, SOX2, and SOX9 in lung tumor samples compared to normal tissues. (**D**–**F**) Five-year survival curves generated from KM plotter show that high expression of CDK9, SOX2, and SOX9 correlate with poor overall survival in lung adenocarcinoma patients. (**G**) Schematic showing the potential mode of action of CDK9 inhibitors to prevent tumor growth. Cyclin T/CDK9 in response to oncogenic signaling leads to its association with BRD4 and recruitment to target promoters. This results in phosphorylation of RNA Pol II CTD, and active transcription of oncogenic genes. Inhibitors targeting CDK9 will interfere with Pol II CTD phosphorylation and active transcription of genes to prevent tumor growth.

Given the above results, we examined the TCGA database to assess if the expression of CDK9, Sox2, or Sox9 were altered in lung adenocarcinomas. As shown in the box plots in Figure 8A–C, tumor samples showed a significantly higher expression of CDK9, SOX2, and SOX9 compared to the normal samples. Survival analysis using the Kaplan Meier (KM) plotter for lung adenocarcinoma showed that high expression of *CDK9*, *SOX2*, *SOX9,* and *MCL1* (*MCL1* survival curve is not shown) was significantly associated with worse survival in the patients (OS, CDK9: *p* = 0.00029; SOX2: *p* = 0.0017; SOX9: *p* = 0.048; MCL1: *p* = 0.00016) (Figure 8D–F) [68]. These observations, together with our results presented here, suggest that inhibiting CDK9 would suppress tumor-initiating embryonic stem cell transcription factors and survival proteins like Mcl1, to overcome NSCLC growth and drug resistance (Figure 8G, schematic).

## 4. Discussion

Lung adenocarcinomas display mutations in several genes, especially K-Ras and EGFR. For EGFR mutant tumors, targeted therapies have shown considerable efficacy, but the patients invariably develop resistance by acquiring additional mutations in the EGFR gene or through activation of alternate signaling pathways, thus developing resistance to the original targeted therapies. Therefore, drugs targeting multiple signaling cascades simultaneously might be more effective in suppressing tumor growth. The success of chemotherapeutic agents that target common cellular functions like DNA replication, microtubule dynamics, and cell cycle progression are a testament to such approach, despite the side effects of such drugs. The best-case scenario would be to have a treatment strategy that suppresses tumor growth by inhibiting tumor promoting signaling cascades and activating tumor suppressive pathways, with minimal side effects. The data presented here on NSCLC cell lines and organoids suggest that such an effect could be achieved by inhibiting CDK9. This is evident from the effect of CDK9 inhibitors on both K-Ras mutant and EGFR mutant lung cancer cell lines. Interestingly, the CDK9 inhibitors were effective against cells that are sensitive or resistant to the newly developed K-Ras_G12C_ inhibitor AMG510 and the third generation EGFR inhibitor, Osimertinib.

CDK9 is known to play a major role in general transcription, and more importantly in transcription driven by oncogenic regulators, such as super enhancers (SE) and transcription factors. Specifically, cancer cells are highly dependent on transcription driven by super enhancers, developing the concept ‘transcriptional addiction’, and such cells are expected to be more sensitive to therapies targeting the transcriptional kinases. Our results show that CDK9 inhibition significantly reduces the viability of K-Ras mutant (A549, H460, H23) and EGFR mutant (H1650, H1975, PC9) lung cancer cell lines at very low nanomolar to less than 0.5 µM concentration of CDK9 inhibitors, namely, AZD4573, LY2857785, and SNS032, respectively. Some of the genes regulated by SE-mediated transcription include *SOX2*, *SOX9*, and *MYC*. Expression of Sox2 and Sox9, the early embryonic transcription factors, are implicated in cancer stem cells (CSCs) or tumor initiating cells. Studies from lung cancers have shown that expression of these transcription factors correlate with survival, growth, therapy resistance, and recurrence of metastatic tumors. Analysis of cells treated with AZD4573 showed that the levels of Sox2 and Sox9 are reduced, implying that inhibiting CDK9 might prevent their tumor promoting functions. C-Myc on the other hand was not reduced significantly, raising the possibility that persistent c-Myc expression could potentially support continued tumor growth. This possible scenario led us to test the efficacy of CDK9 inhibitors in the presence of the BRD4 inhibitor JQ1. While JQ1 by itself showed IC_50_s in the micromolar range (2.5 µM to >10 µM) in majority of the cell lines tested, inclusion of sublethal doses of JQ1 (0.5 µM) significantly improved the efficacy of CDK9 inhibitors on the cancer cells, supporting the contention that either targeting CDK9 alone, or together with BRD4 would potentially be efficacious in combating NSCLC.

Since one of the major impediments in cancer therapy is the development of resistance, we examined if CDK9 inhibitors are capable of eliminating drug-resistant cells. Experiments with Osimertinib-resistant PC9 and AMG510 insensitive H23 and H358 KRAS_G12C_ cells show that CDK9 inhibitors elicit almost identical efficacy on the sensitive and resistant cancer cells. This is a significant result and imply that the CDK9 inhibitors could potentially be effective in NSCLC patients who develop resistance towards targeted therapies.

The potential of a CDK7 inhibitor, THZ1, to overcome Osimertinib resistance, has been described earlier [69]. The authors found that EMT-induced resistance to Osimertinib could effectively be overcome by THZ1. It is likely that the CDK9 inhibitors we tested could be overcoming EMT-mediated resistance, in addition to resistance induced by other means, including the activation of alternate survival signaling events. Similar to the induction of EMT, cell cycle regulatory pathways have been found to be altered in Osimertinib-resistant cells [70,71]; inhibitors of cell cycle progression could overcome such resistance. Induction of stemness and autophagy are other mechanisms that confer resistance to Osimertinib [72]. The suppressive effect of CDK9 inhibitors on these and other relevant pathways to overcome Osimertinib resistance should be evaluated in future studies.

As far as resistance to AMG510 is concerned, several recent studies have identified multiple underlying mechanisms; these have been recently reviewed [73]. One of the common mechanisms of resistance is the reactivation of downstream MAPK pathways after exposure to the inhibitor. It has been suggested that pathways that inhibit MAPK activity are suppressed upon K-Ras G12C inhibition; these include the transcriptional suppression of genes like SPRY, DUSP, and PHLDA, resulting in bypass signaling through the MAPK pathway [74]. It has also been demonstrated that inhibition of K-Ras G12C can lead to the elevated expression of wild type GTP bound Ras, which is insensitive to the inhibitor [75]. This induction is thought to involve EGF signaling events, which can also directly enhance MAPK levels. In addition, single-cell RNA-Seq experiments have suggested that inhibition of K-Ras G12C leads to the accumulation of cells that express mutant or wild type Ras that are in the active GTP-bound state, and are thus resistant to the inhibitor [76]. Thus, activation of bypass MAPK signaling is one of the mechanisms by which cells acquire resistance to AMG510. It can be imagined that the transcriptional component of the bypass signaling machinery are sensitive to CDK9 inhibitors, thus overcoming the resistance to K-Ras G12C inhibitors. The identities of the genes that are targeted by the CDK9 inhibitors remain to be elucidated in future studies.

In addition, activation of the CDK4/6 cell cycle regulatory pathway, which results in proliferation and survival, has been proposed as a survival mechanism against K-Ras G12C inhibition. Supporting this contention, CDK4/6 inhibitors have been shown to overcome resistance to the G12C inhibitors [77,78]. Further, in addition to the cancer cell autonomous mechanisms, the immune system is also thought to contribute to the development of resistance [79]. While the studies presented in this manuscript do not involve the immune system, it is highly likely that CDK9 inhibitors might enhance the anti-tumor immune response, helping to overcome the resistance to the G12C inhibitors.

Another important and novel finding in this study is the increase in p53 levels in NSCLC cell lines upon treatment with the CDK9 inhibitors. This was clearly evident in WT p53 expressing, K-Ras mutant cell lines, such as A549 and H460. H1650 has been described to have homozygous p53 mutation, and we did not observe high levels of p53 or alterations in p53 levels in these cells upon CDK9 inhibitor treatment. This raises some interesting hypotheses: (1) CDK9 inhibition promotes p53-specific signaling and tumor suppression in WT p53 expressing NSCLCs; and (2) mutant p53 expressing NSCLCs respond to CDK9 inhibition in a p53-independent manner. Supporting this hypothesis, both PC9 and H23 cells expressing the mutant p53 also did not show marked alteration in p53 expression upon CDK9 inhibitor treatment. The results from H23 and H1650 cells, together with A549 and H460 cells, suggest that CDK9 inhibitors can induce apoptosis through p53-dependent and independent mechanisms. It is highly likely that the suppression of the anti-apoptotic Bcl2 family member Mcl1 contributes to the induction of apoptosis. The anti-apoptotic proteins Mcl1 and XIAP are known targets of CDK9, and suggests that CDK9 inhibition brings about its effects by suppressing anti-apoptotic or pro-survival signaling, while simultaneously activating pro-apoptotic and tumor-suppressive pathways. Frequency of Mcl1 gain has been shown to be high in NSCLCs and its inhibition has been associated with suppression of tumor growth in mouse models of lung adenocarcinoma [80]. As depicted in the schematic in Figure 8G, inactive CDK9 associated with the 7SKsnRNP complex is activated in response to oncogenic signaling events. BRD4 recruits active P-TEFb complex to promoters of target genes, resulting in RNA Pol II CTD phosphorylation and transcriptional activation. CDK9 inhibitors prevent the CTD phosphorylation, preventing the expression of oncogenic genes like Sox2, Sox9, and Mcl1, resulting in growth inhibition.

Examination of the TCGA database show that CDK9 expression is elevated in lung adenocarcinomas and high expression of CDK9, SOX2, and SOX9 correlates with poor overall survival. The data presented here clearly show that CDK9 inhibitors suppress the expression of Sox2 and Sox9, and interfere with anchorage independent growth of tumor cells. The inhibition of anchorage independent growth is of specific interest as this may suggest that CDK9 inhibitors will be able to interfere with steps involved in tumor dissemination and metastasis. Further, tumors are highly heterogeneous, and tumor growth is supported by signaling from the various cell populations present in the tumor microenvironment. Organoids are the most relevant in vitro system that recapitulates in vivo tumors and this system enable us to test efficacy of a therapy prior to in vivo testing. The results from tumor organoids imply that CDK9 inhibitors are effective in eliminating the heterogeneous cell population in the organoids. Additionally, results from the drug-resistant cells suggest that CDK9 inhibitors, alone or in combination with sublethal doses of BRD4 inhibitor, would be efficacious for patients who are burdened with K-Ras or EGFR mutated lung adenocarcinomas and those who have developed therapy resistance.

## 5. Conclusions

We envisage that inhibitors targeting CDK9 would provide significant anti-tumor activity, as a single agent or in combination with other established therapies, in NSCLC. Two of the CDK9 inhibitors used in our studies, SNS032 and AZD4573, have been in clinical trials for hematological malignancies. While both of these inhibitors show strong inhibitory effect on CDK9, AZD4573 has been reported to be more specific for CDK9. SNS032 has been shown to inhibit other CDKs such as CDK2 and CDK7, and LY2857785 has been shown to affect CDK8 and to a lesser extent CDK7. AZD4573 has been reported to show greater than 10-fold selectivity towards CDK9 compared to other CDKs. Phase I clinical trials conducted with intravenously administered SNS032 has demonstrated limited, target specific activity in multiple myeloma (MM) and chronic lymphocytic leukemia (CLL) patients [81]; there are no clinical data available with LY285778. With AZD4573 currently in multiple clinical trials with relapsed or refractory hematological malignancies, we expect that once the in vivo efficacy is established for AZD4573 in animal models, it could easily be translated to the clinic for testing in lung adenocarcinoma patients.

## Data Availability

The study did not report any data.
